# Preoperative Albumin-to-Alkaline Phosphatase Ratio as an Independent Predictor of Lymph Node Involvement in Penile Cancer

**DOI:** 10.3390/medicina60030414

**Published:** 2024-02-28

**Authors:** Antonio Tufano, Luigi Napolitano, Biagio Barone, Gabriele Pezone, Pierluigi Alvino, Simone Cilio, Carlo Buonerba, Giuseppina Canciello, Francesco Passaro, Sisto Perdonà

**Affiliations:** 1 Department of Maternal-Infant and Urological Sciences, Policlinico Umberto I Hospital, “Sapienza” Rome University, 00161 Rome, Italy; 2 Urology Unit, Department of Neurosciences, Reproductive Sciences and Odontostomatology, University of Naples “Federico II”, 80131 Naples, Italybiagio.barone@aorncaserta.it (B.B.);; 3 Department of Public Health, University of Naples “Federico II”, 80131 Naples, Italy; 4 Associazione O.R.A.—Oncology Research Assistance, 80049 Somma Vesuviana, Italy; 5 Department of Urology, Istituto Nazionale Tumori IRCCS Fondazione G. Pascale, 80131 Naples, Italy

**Keywords:** penile cancer, penile neoplasms, penile surgery, albumin-to-alkaline phosphatase ratio, biomarkers, serological markers

## Abstract

*Background and Objectives:* To investigate the role of preoperative albumin-to-alkaline phosphatase ratio (AAPR) in predicting pathologic node-positive (pN+) disease in penile cancer (PC) patients undergoing inguinal lymph node dissection (ILND). *Materials and Methods*: Clinical data of patients with squamous cell carcinoma (SCC) PC + ILND at a single high-volume institution between 2016 and 2021 were collected and retrospectively analyzed. An AAPR was obtained from preoperative blood analyses performed within 30 days from their scheduled surgery. A ROC curve analysis was used to assess AAPR cutoff, in addition to the Youden Index. Logistic regression analysis was utilized for an odds ratio (OR), 95% confidence interval (CI) calculations, and an estimate of pN+ disease. A *p* value < 0.05 was considered to be as statistically significant. *Results*: Overall, 42 PC patients were included in the study, with a mean age of 63.6 ± 12.9 years. The AAPR cut-off point value was determined to be 0.53. The ROC curve analysis reported an AUC of 0.698. On multivariable logistic regression analysis lymphovascular invasion (OR = 5.38; 95% CI: 1.47–9.93, *p* = 0.022), clinical node-positive disease (OR = 13.68; 95% CI: 4.37–43.90, *p* < 0.009), and albumin-to-alkaline phosphatase ratio ≤ 0.53 (OR = 3.61; 95% CI: 1.23–12.71, *p* = 0.032) were predictors of pN+ involvement. *Conclusions*: Preoperative AAPR may be a potentially valuable prognostic marker of pN+ disease in patients who underwent surgery for PC.

## 1. Introduction

Penile cancer (PC) is a rare malignancy with an incidence of 0.4–0.6% in the United States and Europe [1]. Squamous cell carcinoma (SCC) represents the most common histology, generally occurring in men between 50 and 70 years old, and it is often localized to the glans.

Several risk factors have been demonstrated to elevate the likelihood of developing the invasive disease. Specifically, phimosis, lack of circumcision, obesity, smoking, UVA phototherapy, and socioeconomic status, as well as HPV infection and immune-compromised states represent the potential contributing factors for the development of PC [2].

To date, surgery stands as the primary treatment for PC. However, for precancerous lesions or early-stage malignancies, less invasive approaches, such as topical chemotherapy with imiquimod or 5-fluorouracil (5-FU), laser therapy, or brachytherapy may be considered, since radical treatments may exhibit profound negative impact on the patient’s sexual function, quality of life (QOL), social interactions, self-image and self-esteem [3].

Lymph node involvement is a key prognostic factor in penile SCC, and metastatic disease is typically considered incurable [4]. Preoperative variables such as pathological stage, grade, perineural, and lymph–vascular invasion have been identified as predictors of inguinal LNI.

In this context, the correlation between systemic inflammation has been established as a contributing factor of tumor aggressiveness, with a variety of preoperative serum markers that are currently used in clinical practice [5,6,7]. In this scenario, albumin-to-alkaline phosphatase ratio (AARP) represents a newly developed serum biomarker-based index. AAPR has revealed its prognostic significance in different malignances as well as pancreatic ductal adenocarcinoma, lung, and hepatocellular carcinoma. Moreover, according to previous studies, AAPR is correlated with tumor cell proliferation and inflammation reactions [8,9]. However, no research has ever evaluated the prognostic value of AAPR in patients with PC. Therefore, in this study, we attempted to explore the predictive role of preoperative AAPR in detecting inguinal LNI in patients with SCC treated with excision of the primary tumor + inguinal lymph nodes dissection (ILND).

## 2. Materials and Methods

### 2.1. Study Design

A retrospective analysis of a prospective recorded dataset was screened to identify patients with PC patients treated at our institution, IRCCS Hospital “G. Pascale” of Naples, Italy, between January 2016 and October 2021. The research was conducted according to the Declaration of Helsinki, on ethical principles for medical research involving human subjects. All patients provided written informed consent for the inclusion of their data in a database and the use for scientific research purposes. The ethical committee was informed of the non-experimental design of the retrospective investigation and endorsed the study.

The inclusion criteria were as follows: (1) primary SCC PC excised surgically with bilateral ILND following a modified or radical ILND template, and (2) tumor pathology confirmed by an expert uro-oncology pathologist. Exclusion criteria from the study were: (1) the presence of pelvic lymph node involvement or presence of distant metastasis at diagnosis; (2) patients with a low risk of nodal tumor involvement, in which ILND was not indicated; (3) patients lacking the specified complete information; (4) patients with histology other than SCC; and (5) patients with a history of prior radiotherapy or neoadjuvant chemotherapy were excluded from the analysis.

The baseline and clinical characteristics of patients were collected by trained personnel from medical records and by routine venous-blood samples obtained within 30 days before surgery. When there were multiple reports available, the analysis focused on the report conducted nearest to the surgery date. The TNM stage was defined according to the 8th edition (2016) of the TNM staging system. Cases prior to 2016 were reclassified according to the 8th edition.

The data collected included age, primary tumor surgery, ASA score, Charlson Comorbidity Index, tumor grade, pathologic T stage, lymphovascular invasion, perineural invasion, high-risk human papillomavirus co-infection, clinical and pathologic N stage, surgical margins, and preoperative AAPR. The AARP was calculated by dividing the serum albumin level (g/L) by serum alkaline level (U/L). We therefore divided patients into two cohorts based on the APPR cut-off obtained.

### 2.2. Statistical Analysis

Means and standard deviations were reported for continuous variables while frequencies and percentages were reported for categorical variables. A Kolmogorov–Smirnov test was used to assess normality of data before proceeding to further analysis. A Mann–Whitney U Test was utilized to evaluate continuous variables, while Chi-square test was utilized for categorical variables analysis. Receiver operating characteristic (ROC) curve analysis was used to assess AARP cutoff, in addition to the Youden Index. Logistic regression analysis was utilized for odds ratio (OR), 95% confidence interval (CI) calculations, and to estimate pathologic node-positivity. Statistical analysis was conducted using IBM SPSS software (version 25, IBM Corp, Armonk, NY, USA), A *p* value < 0.05 was considered to be statistically significant.

## 3. Results

**Figure 1:**
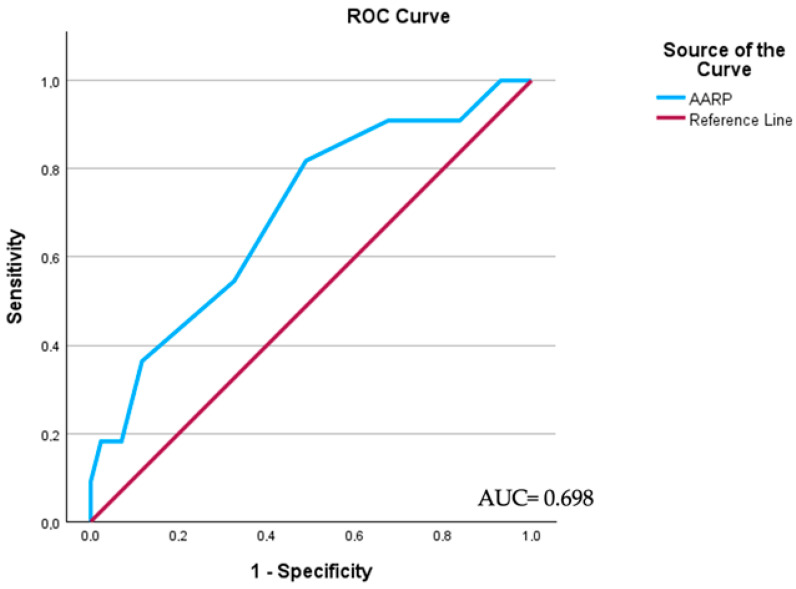
Receiver operating characteristic (ROC) curve and area under the curve (AUC) to define the optimal AAPR cutoff. AUC: 0.698 (*p* = 0.045).

Data concerning the 42 PC patients were collected and included in the study, with a mean age of 63.6 (±12.9) years. Overall, 30 (71.4%) had confirmed inguinal LNI (pN+) after radical or modified ILND. Descriptive characteristics and perioperative data of the whole cohort are reported in Table 1. According to ROC curve analysis, which reported an AUC of 0.698 (*p* = 0.045), we defined a cut-off set at 0.05, according to the Youden Index (Figure 1). The cut-point value of AAPR was determined to be 0.53. A total of 18 (42.9%) patients had an AAPR ≤ 0.53, whereas 24 (57.1%) patients exhibited an AAPR > 0.53. No statistically significant differences were reported among baseline characteristics between the two groups (all *p* > 0.05), despite clinical and pathological N stage (*p* = 0.03 and *p* = 0.02).

On the univariable logistic regression analysis, the presence of lymphovascular invasion (OR = 4.88; 95% CI: 1.97–8.90, *p* = 0.003), clinical node-positive disease (OR = 16.76; 95% CI: 6.89–39.02, *p* < 0.001) and albumin-to-alkaline phosphatase ratio ≤ 0.53 (OR = 3.91; 95% CI: 1.89–9.71, *p* = 0.014) were independent predictors of pN+ disease (Table 2). Their predictive role was confirmed on multivariable analysis, where lymphovascular invasion (OR = 5.38; 95% CI: 1.47–9.93, *p* = 0.022), clinical node-positive disease (OR = 13.68; 95% CI: 4.37–43.90, *p* < 0.009), and albumin-to-alkaline phosphatase ratio ≤ 0.53 (OR = 3.61; 95% CI: 1.23–12.71, *p* = 0.032) reached statistical significance (Table 2).

## 4. Discussion

Despite the potentially life-saving recommendations, trends still indicate a suboptimal adherence to PC guidelines [10,11]. Establishing novel prognostic indicators is crucial for a correct risk stratification of PC patients, providing valuable guidance for clinical management decisions and follow-up strategies [12,13]. In this scenario, several systemic inflammation biomarkers, including C-reactive protein (CRP), platelet-to-lymphocyte ratio, lymphocyte-to-monocyte ratio, and neutrophil-to-lymphocyte ratio have been proposed as potential prognostic factors in PC [14,15]. AAPR is a newly developed serum biomarker-based index, defined by the straightforward and efficient combination of two serum biomarkers, albumin and alkaline levels. Both markers can be easily and cost-effectively obtained as part of routine clinical practice.

It is widely known that a liver function test serves as a readily available blood test for assessing liver function. Albumin, a protein uniquely synthesized by the liver, is a key component of this evaluation. In patients with hepatocellular carcinoma (HCC), hypoalbuminemia is a result of compromised liver function due to the underlying chronic liver disease. Moreover, albumin is a protein particularly consumed by an aggressive tumor, and hypoalbuminemia may impair the metabolism and the function of immune cells. Hypoalbuminemia is also linked to a sustained systemic inflammatory response, originating either from the tumor itself or as a host reaction [5]. Hence, albumin stands out as a valuable indicator of hepatic protein synthetic capacity and serves as a meaningful marker for the host’s inflammatory response, playing a crucial role in tumorigenesis from tumor initiation to metastatic dissemination [16].

Alkaline phosphatase, primarily found in the liver, bone, and kidney, has frequently been noted to exhibit associations with bone metastasis, as well as liver and kidney diseases [17]. Moreover, alkaline phosphatase possesses anti-inflammatory properties, capable of inhibiting inflammatory reactions, and is linked to the nutritional status [18]. Nevertheless, previous research has demonstrated correlations between alkaline phosphatase levels and the prognosis of esophageal cancer, colorectal cancer, nasopharyngeal cancer, bladder, and renal cancer [19,20]. Alkaline phosphatase seems to be a regulator of immune response, involved in tumor growth regulation, metastasis, and progression. Recent evidence suggested that ALP can serve as a tumor-associated antigen and biomarker of cancer cell proliferation [21].

Interestingly, several studies have reported that AAPR represents a prognostic factor in different malignances as well as hepatoma, cholangiocarcinoma, small cell lung cancer (SCLC), non-small cell lung cancer (NSCLC), and renal cell carcinoma. Ren et al., in a recent systematic review including eight studies and 3271 patients, reported that AAPR is an unfavorable factor in patients with surgically treated urological cancers, and low AAPR was associated with inferior survival [22]. Hu et al. reported that in 648 patients with surgically treated non-metastatic renal cell carcinoma, low AAPR was an unfavorable prognostic factor [23]. Moreover, Won et al. investigated the prognostic value of AAPR in patients with non-metastatic RCC treated with partial or radical nephrectomy [24]. Their data suggest that AAPR could be a promising prognostic factor for prediction of recurrence and survival in patients with non-metastatic RCC who undergo nephrectomy [24]. Nevertheless, Yoshino et al. in a retrospective study showed that AAPR may be significantly associated with the outcome of nivolumab monotherapy in mRCC patients [25]. Despite this evidence, no relevant data reported the association of AAPR and LNI in PC patients. LNI is associated with poor survival, and early diagnosis and management significantly impacts survival and the quest for validated molecular markers or precise minimally invasive tests capable of identifying nodal metastasis remains a priority.

Our results confirmed the role of AAPR in the prediction of LNI on both univariable and multivariable analysis (OR = 3.91 and OR = 3.61, respectively). However, we can consider our study as an initial experience regarding the role of AAPR as a prognostic biomarker in PC. Furthermore, prospective and large-scale studies are warranted to validate our findings.

This study presents some limitations: First, the retrospective and single-center nature of the study and the relatively small sample size of patients might result in biases related to the patient’s selection and treatment. Second, the determination of the AAPR value is influenced significantly by the race and age of the patients. Third, factors such as non-explored comorbidities and the extent of systemic inflammation within the study’s population may also play a role in contributing to this value.

## 5. Conclusions

In conclusion, low preoperative AAPR could represent an independent predictor of LNI in patients with PC. Considering the limitations, AAPR is an inexpensive, easy and convenient test to use in the current clinical practice to predict the prognosis, risk stratification, to guide the treatment, and follow up for patients.

## Figures and Tables

**Table 1:** Characteristics of penile cancer patients included.

	Overall	AAPR ≤ 0.53	AAPR > 0.53	*p* Value
	*n* = 42	*n* = 18	*n* = 24	
**Age, mean (SD)**	63.6 (12.9)	65.3 (13.7)	62.1 (11.3)	0.46
**Smoking, *n* (%)**				
Never	8 (19.1)	3 (16.7)	5 (20.8)	0.26
Current	14 (33.3)	6 (33.3)	8 (33.3)	
Former	20 (47.6)	9 (50.0)	11 (45.9)	
**HPV, *n* (%)**				
High Risk	19 (45.2)	7 (38.9)	12 (50.0)	0.49
Intermediate-Low Risk	1 (2.4)	1 (5.6)	0 (0.0)	
No	4 (9.5)	2 (11.1)	2 (8.3)	
Unknown	18 (42.9)	8 (44.4)	10 (41.7)	
**Diabetes, *n* (%)**	8 (19.0)	3 (16.6)	5 (20.8)	0.38
**Hypertension, *n* (%)**	18 (42.8)	7 (38.8)	11 (45.8)	0.56
**ASA score, median (IQR)**	2 (2–2)	2 (2–2)	2 (2–2)	0.78
**Charlson Comorbidity Index, median (IQR)**	3 (2–4)	3 (2–4)	3 (2–4)	0.87
**Primary tumor surgery, *n* (%)**				
Total penectomy	7 (16.7)	3 (16.7)	4 (16.6)	0.23
**Partial Penectomy**	21 (50.0)	8 (44.4)	13 (54.2)	
Penile-sparing surgery	14 (33.3)	7 (38.9)	7 (29.2)	
**Clinical N stage, *n* (%)**				
cNx	1 (2.4)	1 (5.5)	0 (0)	**0.03**
cN0	18 (42.8)	5 (27.8)	13 (54.2)	
cN+	23 (54.8)	12 (66.7)	11 (45.8)	
**Pathologic T stage, *n* (%)**				
pTa/T1	7 (16.7)	3 (16.7)	4 (16.7)	0.08
pT2	13 (30.9)	5 (27.8)	8 (33.3)	
pT3	22 (52.4)	10 (55.5)	12 (50.0)	
pT4	0 (0.0)	0 (0.0)	0 (0.0)	
**Primary tumor grade, *n* (%)**				
G1/G2	16 (38.1)	7 (38.9)	9 (37.5)	0.88
G3/G4	26 (61.9)	11 (61.1)	15 (62.5)	
**Pathologic N stage, *n* (%)**				
pN0	12 (28.6)	3 (16.7)	9 (37.5)	**0.02**
pN+	30 (71.4)	15 (83.3)	15 (62.5)	
**Lymphovascular invasion, *n* (%)**				
No	19 (45.2)	8 (44.4)	11 (45.8)	0.67
Yes	23 (54.8)	10 (55.6)	13 (54.2)	
**Perineural invasion, *n* (%)**				
No	11 (26.2)	5 (27.8)	6 (25.0)	0.38
Yes	13 (30.9)	6 (33.3)	7 (29.2)	
Unknown	18 (42.9)	7 (38.9)	11 (45.8)	
**Positive Margins, *n* (%)**	0 (0.0)	0 (0.0)	0 (0.0)	-

**Table 2:** Logistic regression model predicting pathologic inguinal node-positive disease (pN+).

Variable	Univariable Analysis				Multivariable Analysis			
	OR *	95.0% CI *			OR *	95.0% CI *		
		Lower	Higher	*p* Value		Lower	Higher	*p* Value
**Primary tumor grade**								
G1/G2	Ref.	-	-	-	-	-	-	-
G3/G4	1.73	0.78	2.1	0.42				
**Lymphovascular Invasion**								
No	Ref.	-	-	-	Ref.	-	-	-
Yes	4.88	1.97	8.90	**0.003**	5.38	1.47	9.93	**0.022**
**Clinical N stage**								
cN0	Ref.	-	-	-	Ref.	-	-	-
cN+	16.76	6.89	39.02	**<0.001**	13.68	4.37	43.90	**0.009**
**Albumin-to-Alkaline phosphatase ratio**								
>0.53	Ref.	-	-	-	Ref.	-	-	-
≤0.53	3.91	1.89	9.71	**0.014**	3.61	1.23	12.71	**0.032**

* OR = odds ratio; CI = confidence interval.

## Data Availability

The data presented in this study are available upon request from the corresponding author. The data are not publicly available due to privacy restrictions.
